# A Vocal-Based Analytical Method for Goose Behaviour Recognition

**DOI:** 10.3390/s120303773

**Published:** 2012-03-21

**Authors:** Kim Arild Steen, Ole Roland Therkildsen, Henrik Karstoft, Ole Green

**Affiliations:** 1 Department of Engineering, Aarhus University, Finlandsgade 22, 8200 Aarhus N, Denmark; E-Mails: hka@iha.dk (H.K.); ole.green@agrsci.dk (O.G.); 2 Department of Bioscience, Aarhus University, Grenåvej 14, 8410 Rønde, Denmark; E-Mail: oth@dmu.dk

**Keywords:** support vector machines, goose behaviour, pattern recognition, vocalisations, GFCC

## Abstract

Since human-wildlife conflicts are increasing, the development of cost-effective methods for reducing damage or conflict levels is important in wildlife management. A wide range of devices to detect and deter animals causing conflict are used for this purpose, although their effectiveness is often highly variable, due to habituation to disruptive or disturbing stimuli. Automated recognition of behaviours could form a critical component of a system capable of altering the disruptive stimuli to avoid this. In this paper we present a novel method to automatically recognise goose behaviour based on vocalisations from flocks of free-living barnacle geese (*Branta leucopsis*). The geese were observed and recorded in a natural environment, using a shielded shotgun microphone. The classification used Support Vector Machines (SVMs), which had been trained with labeled data. Greenwood Function Cepstral Coefficients (GFCC) were used as features for the pattern recognition algorithm, as they can be adjusted to the hearing capabilities of different species. Three behaviours are classified based in this approach, and the method achieves a good recognition of foraging behaviour (86–97% sensitivity, 89–98% precision) and a reasonable recognition of flushing (79–86%, 66–80%) and landing behaviour(73–91%, 79–92%). The Support Vector Machine has proven to be a robust classifier for this kind of classification, as generality and non-linear capabilities are important. We conclude that vocalisations can be used to automatically detect behaviour of conflict wildlife species, and as such, may be used as an integrated part of a wildlife management system.

## 1. Introduction

In many parts of the world, damage caused by wildlife creates significant economic challenges to human communities. Since human-wildlife conflicts are increasing [1] the development of cost-effective methods for reducing damage or conflict levels is important in wildlife management. A wide range of devices to detect and deter animals causing conflict are used in wildlife damage management, although their effectiveness is often highly variable [2]. Present scaring devices are often activated electronically, through detection of motion and/or body heat (e.g., infrared sensors, Gilsdorf *et al.* [2]). In most cases scaring devices are non-specific, so they can be activated by any animal, not only when individuals of the target species enters the area. This increases the risk of habituation, which is often the major limitation on the use of scaring devices [3]. Although random or animal-activated scaring devices may reduce habituation and prolong the protection period over non-random devices [3], to our knowledge no cost-effective concept circumventing the problems of habituation has yet been developed.

**Figure 1.**
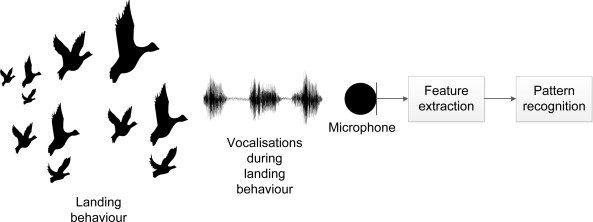
Concept of classification of landing behaviour, based on recorded vocalisations.

For our purpose, we identified three relevant behaviours (landing, foraging and flushing), which are all accompanied by distinct vocalisations easily identified by the human ear. The vocalisations allow us to identify a flock of geese (1) attempting to land; (2) foraging or (3) being flushed. By using vocalisation recognition, we are then able to automatically detect a flock of geese attempting to land and to assess the effect of a scaring (see Figure 1). Thereby, the concept allows us to monitor potential habituation (*i.e.*, the situation, when geese no longer respond to scaring) and, accordingly, change our scaring strategy.

Typical methods used within animal behaviour research are based on attached tracking devices, like Global Positioning System (GPS) [4] or other wireless transmitters in a wireless sensor network [5,6], or accelerometers, measuring the movement of specific parts of the animal body [7]. Acoustic information has also been used in chewing behaviour recognition of cows [8], however these methods also rely on attaching a device on the animals. These methods are not suitable when the purpose of the animal behaviour recognition, is to utilize the results in a wildlife management system, as it is not possible to attach these devices on the animals. Vallejo and Taylor [9] uses vocalisations for source identification, based on a microphone array and thereby recognise bird behaviour, however the link between a specific vocalisation and behaviour is not found. Recognition of vocalisation, however does provide a method for behaviour recognition without the need to attach any devices on the free-living animals.

Recently, audio processing and pattern recognition methods have been used for recognition of animal vocalisations [10–13] and behaviour [14–17], in a controlled experiments or on single animals. This research within automatic vocalisation recognition has been highly influenced by methods conducted within human speech and speaker recognition. This includes feature extraction techniques, focused on cepstral features [18,19] and pattern recognition algorithms such as Hidden Markov Models (HMMs) [20,21], Gaussian Mixture Models (GMMs) [20] and Support Vector Machines (SVMs) [9,22,23].

The Mel Frequency Cepstral Coefficients (MFCC) have proven to be good features within human speech recognition, as they model the human perception of sound, and is therefore also widely used within animal vocalisation recogntion. However, animal sound perception may be different than human sound perception, and other features may be more suitable. In this paper, Greenwood Function Cepstral Coefficient (GFCC) features are used as features, to describe the vocalisations, as they, like MFCC, model the preception of sound, but can be adjusted to the hearing capabilities of different species [24].

The SVM is a supervised learning algorithm which can be used in both linear and non-linear pattern recognition problems [25]. The models are based on a structural risk minimisation principle, which improves the generalisation ability of the classifier [26]. Since the introduction of the model in the 1990s [27], the SVM has become a popular method of choice for many applications, including behaviour recognition, speaker identification and object recognition [23,28,29]. In our research, the SVM was used in a multiclass classification task to classify one of three behaviours, based on their vocalisations. The models were trained with labeled data, which were extracted from the recordings.

This paper presents a new concept for detection of animal behaviour based on its vocalisation. Methodologies developed for speech recognition have been adjusted and used to distinguish between three specific behaviours. The analytical method, described in this paper, is part of ongoing research regarding a system capable of detecting behaviour of conflict species, such as barnacle goose (*Branta leucopsis*), and adjust its scaring stimuli based on the detected behaviour in order to avoid habituation.

## 2. Materials and Methods

This section describes the chosen study species, the location of recording and the methods applied.

### 2.1. Study Species

We chose the Russian/Baltic population of barnacle geese as our study subject. The dramatic increase in this population over the past few decades has led to serious conflict between agriculture and geese throughout the wintering range. In Denmark, the large flocks of barnacle geese, which occur along the west coast until late spring, are causing damage to both winter cereals and pastures. Moreover, barnacle geese, like other goose species, are vocal and therefore suitable for studying the relationships between vocalisations and behaviour. Although various methods have been employed to scare barnacle geese off agricultural land, to date, no successful long-term, cost-effective scaring method has been found.

### 2.2. Study Site

Vest Stadil Fjord is situated on the west coast of Jutland (56°11′26.23″*N*, 8^°^7′39.07″*E*) surrounded by cereal fields, pastures, marsh and reed beds. Vest Stadil Fjord is an important staging and wintering area for both the Svalbard-breeding population of pink-footed geese (*Anser brachyrhyncus*) and the Russian-breeding barnacle geese.

The recordings took place in April 2011, when up to 10,000 barnacle geese staged in the area.

### 2.3. Equipment

**Figure 2.**
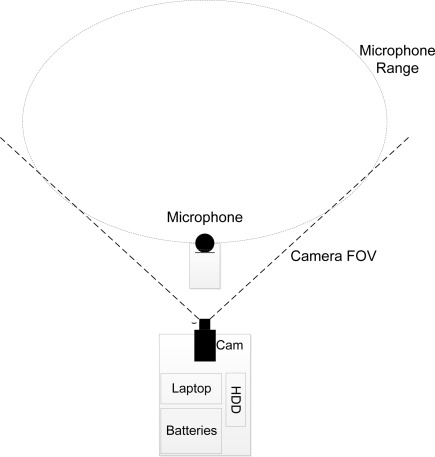
Sketch of the equipment used for data collection. The camera captures a video stream for later inspection of behaviour. Both audio and video data are stored on an external hard drive. The dashed lines indicate microphone range and camera field of view.

A combination of a shielded shotgun microphone (Sennheiser MKE 400) and a machine vision camera (uEye UI-1245LE-C) with a field of view (FOV) of 45° connected to a laptop were used for recordings. A multiple-shielded audio extension cable was used to minimise loss in fidelity. The camera and laptop were placed in a box at the edge of the field, whereas the microphone was placed 10 m in front of the camera, closer to the geese. The system was powered by two 12 V 92 Ah deep cycling car batteries and data were stored on 3 TB external hard drive. An overview is seen in Figure 2 (a detailed description can be found in Steen *et al.* [30]).

### 2.4. Data Collection

The vocalisations where recorded with a sample rate at 44.1 kHz. An uncompressed audio file (wave) was saved every five minutes during daylight hours.

The synchronised audio and video recordings were stored on an external hard drive for later processing. In order to capture the movements of the geese, the video stream was recorded at a frame rate of 20 frames per second.

During the study period there were two occurrences of barnacle geese, at two different dates, within the FOV of the camera. The recordings were categorised into the three behaviours of interest: landing, foraging and flushing. In Table 1, a description of the behaviours and the duration of the recordings are listed. The behaviours were observed as single events in both days, where the behaviours occoured.

The behaviours were manually labeled and observations, where the fidelity of the audio recordings were below a certain threshold, were excluded. The selected audio sequences were divided into 100 ms sequences.

The short duration of the recordings of the behaviour flushing results from the fact, that this event only covers a short time span.

### 2.5. Support Vector Machines

One of the most popular pattern recognition algorithms used in both human speech and animal vocalisations recognition is HMM, because of its capability to model both stochastic and temporal variations [10]. However, in the case of classification of flock behaviour, the vocalisations, produced by the flock, looses the temporal information, as multiple geese vocalise at the same time. Lately SVM models have been used in bird species recognition research [9,22], and other research working with real-world classification tasks [28,29]. SVM models are able to handle non-linear classification tasks, and they are based on structural risk minimisation principle, which improves the generalisation ability of the classifier [26]. For these reasons, the SVM has been chosen in this research.

Given *n* training examples {*x_i_, y_i_*}, *i* = 1 . . . *n*, where *x_i_* is the *i*th feature vector of the training set and *y_i_* ∈ {−1*,* 1} is the class label of the *i*th feature vector, the SVM model is trained to find a hyperplane Equation (1) which maximizes the margin (1/ ‖**w**‖) between two linearly separable labeled data sets. The hyperplane is parametrized by the weights **w** and the bias b.

(1) $$f(x)=w^{T}\;x+b$$

This represents a binary classification problem, however, SVMs can also be used in multiclass problems as: one-*versus*-all SVMs, one-*versus*-one SVMs, pairwise coupling and error-correcting output code SVMs [26].

Maximizing the margin 1/ ‖**w**‖ is equivalent to minimizing ‖**w**‖^2^, which leads to a constrained optimization problem:

(2) $$\begin{array}{cc} \underset{w,b}{\mathrm{minimize}} & \frac{1}{2}{\left\|w\right\|}^{2}\\ \text{subject to}: & y_{i}(w^{T}\;x_{i}+b)\geq 1\;\;\;i=1,\ldots ,n \end{array}$$

where the Lagrangian multiplier can be used to solve this. The Lagrangian function is defined as

(3) $$L(w,b,a)=\frac{1}{2}{\left\|w\right\|}^{2}-\sum_{n=1}^{N}a_{n}\left\{y_{n}(w^{T}\;\phi (x_{n})+b-1\right\}$$

where *a_n_* ≥ 0 are the Lagrange multipliers, and *ϕ*(**x**_n_) is a transform function. The transform function is introduced, as real-world data is seldom linearly separable. The function transforms the features into a higher dimension, where they are linearly separable [25]. This is not a computationally costly expansion of the SVM, as kernel function (4) provides that only the dot product needs to be calculated.

(4) $$k(x,\;x^{\prime })=\phi (x^{T})\phi (x^{\prime })$$

Some of the more popular kernel functions are the linear kernels, the radial basis function kernels Equation (5) and the polynomial kernels [25]. In this study, the radial basis function (RBF) is used:

(5) $$k(x,\;x^{\prime })=\exp\left(-\gamma {\left\|x-x^{\prime }\right\|}^{2}\right)$$

where the parameter *γ* controls the kernel radius. Determining this parameter is therefore a part of the SVM training procedure. The optimization problem given in Equation (2) does not allow for misclassification, which may lead to overfitting when training SVM models. Therefore the *soft-margin* SVM was introduced by Cortes and Vapnik in [31], where the constant *C* is introduced. The parameter allows for misclassification in the training of SVM models, and is also used to adjust for differences in data size for each class. A more detailed description of SVM can be found in [25,27].

The solution to the optimization problem in Equation (2) is

(6) $$f(x)=\sum_{n=1}^{N}a_{n}\;y_{n}\;k(x,\;x_{n})+b$$

where the sign of *f*(*x*) is evaluated to recognise the class of new data.

In this study, SVM is used for multiclass classification using the one-*versus*-one method. An SVM is trained for all *K* classes, where the *k*th model, constructs a hyperplane between class *m* and *n*. In our case, this means that each of the three models separates two distinct behaviours.

### 2.6. Acoustic Feature Extraction

The features used to describe animal vocalisation, in a recognition setting, are inspired by the research done within human speech and speaker recognition [19,20]. Here cepstral coefficients, such as the MFCC, are among the most popular [32,33].

The MFCC features are derived from the mel-scale, which is a non-linear frequency mapping adjusted to human hearing capabilities. A mel is a unit of measure of perceived pitch or frequency of a tone. In Fant [34] an approximation is given by

(7) $$F_{\mathrm{mel}}=\frac{1000}{\log(2)}\;\log\;\left(1+\frac{F_{\mathrm{Hz}}}{1000}\right)$$

The calculation of MFCC is often carried out using a mel-scale filter bank, consisting of a number of critical band filters with center frequencies adjusted to the mel-scale [33]. The number of filters in the filter bank depend on the application, and various implementations of MFCC feature extraction have been used in speech recognition tasks [35]. The bandwidth of these applications differ, and as barnacle geese vocalisations contain most of their spectral information in the 500–6000 Hz band [36], it is comparable to the bandwidth used by Davis and Mermelstein [37] in their novel paper from 1980, where 20 filters are used. Therefore, 20 filters are used in the feature extraction of geese vocalisations.

These features have been shown to be useful in human speech recognition [33,38], however animals do not perceive sounds equally as humans, which means that MFCC may not be useful for animal vocalisation feature extraction. In Clemins *et al.* [24] generalized perceptual features are introduced. The feature extraction is based on the Greenwood function [39], which assumes that sound perception is on a logarithmic scale (like the mel-scale), but that this scale differs for different species. Greenwood found this to hold true for mammals, however Adi et al. [40] use GFCC for recognition of ortolan bunting (*Emberiza Hortulana*) songs in Adi *et al.* [40]. The frequency warping function looks similar to the mel-scale warping, and the perceived frequency mapping is calculated as

(8) $$F_{p}=\frac{1}{a}\;\log_{10}\;\left(\frac{F_{\mathrm{Hz}}}{A}+k\right)$$

Here the constants *a*, *A*, and *k* are species specific, however the constants *a* and *A* can be derived from knowing *k*. LePage [41] shows that *k* can be approximated by a value of 0.88, which has been used in this research as well. The constants *a* and *A* can then be derived by knowing the hearing frequency range for the specific species (*f_min_*,*f_max_*), see Equations (9) and (10).

(9) $$A=\frac{f_{\min}}{1-k}$$

(10) $$a=\log_{10}\;\left(\frac{f_{\max}}{A}+k\right)$$

**Figure 3.**
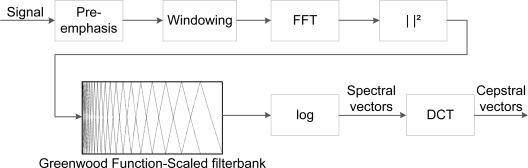
Block diagram of the acoustic feature extraction performed on the recorded vocalisations. A total of 21 features were extracted and six features were chosen based on feature selection techniques.

The calculation of GFCC is illustrated in Figure 3, where the incoming signal has a duration of 46 ms (2048 samples), as cepstral coefficients are derived from short-time analysis. The log-energy of each critical band is represented by spectral vectors, and a cosine transform converts the spectral vectors into cepstral vectors, according to the formula

(11) $$c_{n}=\sum_{k=0}^{K-1}S_{k}\;\cos\;\left(n\left(k-\frac{1}{2}\right)\frac{\pi }{K}\right)\;\;\;\;n=0,\ldots ,K-1$$

Here *c_n_* is the *n*th cepstral coefficients and *S_k_* is the spectral log-energy of the *k*th band. In this research 20 critical band filters were used, which gives a feature vector of dimension 21, as the 0th order cepstral coefficient is included (see Brookes [42]). The filters were hamming shaped, however both hanning and triangle shaped filters are often used in MFCC feature extraction [35].

As SVM models are based on maximizing the margin, the performance of the classifier will decrease if classes have severe overlaps. In the context of this paper, this could be the case if cepstral features does not describe the actual vocalisation, but the random background noise. These features will not provide information about the behaviour, and they could potentially cause class overlaps. Therefore feature selection has utilised to reduce the class overlap.

In this research, the feature selection selects the subset of cepstral coefficients which have the best discriminant capabilities. The feature selection is performed using the *branch and bound* algorithm, which finds the optimal subset of features given that the selection criterion is monotonic [26]. In this research, the sum of squared euclidian distances between features, have been used as the criterion. Using this strategy, six cepstral coefficients were chosen (cepstral coefficient number 16, 15, 5, 4, 3 and 1) and used for training and classification.

### 2.7. Behaviour Classification

**Figure 4.**
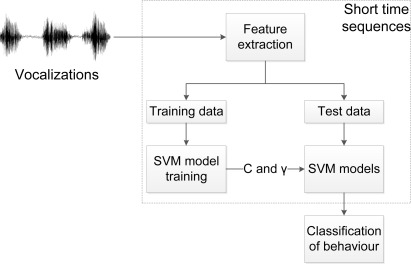
The flow of behaviour classification. The audio data is divided into short time sequences and feature extraction, modeling and classification is performed.

The classification of behaviour is based on the methods described in the two previous sections, and a flow describing the procedure of the behaviour classification in this research, is shown in Figure 4. The vocalisations are divided into short-time sequences, and feature extraction is performed, as shown in Figure 3. The data is divided into training and test data; whereas the SVM models are trained and utilized for behaviour classification. The behaviour classification is based on the entire audio sequence (100 ms is used in this research).

The acoustic feature extraction was performed in MATLAB R2010b, using the Voicebox toolbox [42]. The training and evaluation of the SVMs was performed using LibSVM, which is an open-source SVM toolbox supporting multiple programming languages [43].

The extracted features for the three behaviours were divided into a training data set and a test data set. There were two strategies for evaluation of classifier performance. One was to use data from day 1 as training data and data from day 2 as test data. This test strategy covers the generalisation capabilities of the classifer, as a good performance will indicate good performance on unseen data. The second test mixes all data and perform a 5-fold crossvalidation, using 4*/*5 as training data and the remaining 1*/*5 as test data. This measures the overall performance of the classifier. In the case of using day 1 as training data, the data was divided accordingly (day 1/day 2): flushing (44/56%), foraging (60/40%) and landing (62/38%), due to the distribution of the behaviours in the two days. The two strategies are named Test A and Test B, respectively.

Before training the models, the data was normalised such that all feature vectors had zero mean and unit variance Equation (12), to prevent certain features from dominating classification results due to large numerical values [26].

(12) $$F_{i,j}^{\prime }=\frac{F_{i,j}-\mu_{j}}{\sigma_{j}}$$

The training of the models consists of finding values for C and *γ* (as RBF kernel was chosen). This is done with a grid search, where every combination of C and *γ* is tested, within a predefined range or until a termination criteria is met. The evaluation of C and *γ* values are conducted using a five-fold cross validation scheme [44], where the C and *γ* with the average best cross validation rate is chosen. The grid search is done for all three SVM models, with iterative values of 2^−10^, 2^−9^, . . ., 2^9^, 2^10^ [44]. As more data for foraging and landing behaviour is available, the C values are scaled according to Equations (13) and (14), to compensate for this [45]

(13) $$C_{1}=\frac{N}{2\cdot N_{1}}$$

(14) $$C_{2}=\frac{N}{2\cdot N_{2}}$$

Here N is the total number of feature vectors in the training data and *N*_1_ and *N*_2_ are the number of feature vectors for class one and two.

**Figure 5.**
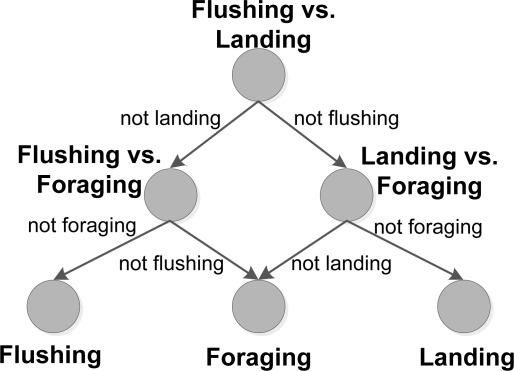
One-*versus*-one classification in a directional graph, where the direction is based on the SVM model results. The binary classification in each node, will result in classification of a single behaviour.

A total of three SVM models were trained, in a one-*versus*-one setup. The classification scheme is seen in Figure 5, where a directional graph [22,46] is used in the classification of behaviour. First the SVM model, modeling the hyperplane between *flushing* and *landing* behaviour, is evaluated and further evaluation steps are based on this result. The classification results are presented in a confusion matrix in the results section (see Table 2), which gives the number of correct positive predictions (as bold numbers) and correct negative predictions, where the classifier rejects a behaviour correctly. Positive predictions or negative predictions, which are incorrect, are also given in the table. The performance of the models are given by three measures: accuracy, precision and sensitivity.

## 3. Results

**Figure 6.**
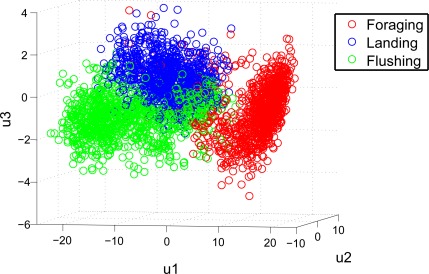
Plot of the first three principal components of the extracted features after feature selection has taken place. It can be seen from the plot, that it is possible to discriminate between the three behaviours, however the vocalisations for landing and fleeing have some similarities.

The GFCC feature extraction makes it possible to discriminate between the vocalisations of the described behaviours. This is visualised in Figure 6, where the three first principal components of the selected features, are shown. The principal components are derived via principal component analysis (PCA) [47], and are the linear combination of the selected features which preserves the most variance in a smaller dimensional space. In Figure 6, it is seen that foraging behaviour seems easiest to discriminate.

This observation is also supported in Table 3, where the overall performance of the classification is described via statistical measurements. The results in Table 3 are derived from the confusion matrix shown in Table 2, and it is seen that the overall classification performance for foraging behaviour is higher than the other two, which is visualised in Figure 6. However the overall classification performance is high, with accuracy measures over 90%. Some variability in precision and sensitivity for Test A and B is present.

The results from Test A show that the SVM models are capable of classifying unseen data, from another day, with high accuracy and precision. In this test the ratio between training and test data was close to 50/50. The results in Test B show the overall performance of the classifier. In this test, the precision was a bit lower for flushing and landing behaviour. This is expected because the vocalisations of the two behaviours are quite similar, which makes it harder for the classifier to give precise results when these behaviours are present in the audio data.

## 4. Discussion

The concept of using behaviour recognition in a wildlife management system requires a precise classification for the detection of goose behavior. Indeed, the results showed that acoustic measurements, feature extraction and statistical modeling may be used to classify their behaviour with a relatively high precision. Although two of the behaviours (*i.e.*, landing and being flushed) have similarities in their vocalisations, the accuracy of classification was more than 90% for all behaviours. Therefore, by combining the three behaviors (*i.e.*, landing, foraging, being flushed), we may obtain information sufficiently accurate for the system to respond appropriately to the presence or absence of geese in the camera FOV.

For instance, foraging behaviour was classified with a very high precision and sensitivity, which may be augmented with sequential information regarding detection of landing behaviour, as foraging behaviour would be a result of landing behaviour. However, this has yet to be investigated specifically. The detection of geese being flushed is also very important in the automatic setup, as this allows the system to verify, whether scaring has been successful or not. The performance of this detection is similar to landing behaviour, however the same argument holds, that the system could accurately use sequential information to provide a more detailed analysis on flushed behavior following a specific scaring stimulus.

In this paper, the recorded data consisted of audio and video data, although the video was only used for manual observation. To further increase classification precision, computer vision algorithms, could be to incorporated to automatically track and classify behaviour. Examples of using computer vision for this can be found in Perner [48] and Matetić *et al.* [49].

In this paper GFCC was used as features, and an attempt to adjust the constants to geese vocalisations has been applied. However, these are based on an approximation of the constant *k*, which might not be true for geese. The authors suggest an optimization based approach to derive the constants to be used, where the criteria could be discriminant analysis. This has yet to be investigated.

The concept of using vocalisation in automatic behaviour recognition could easily be incorporated in other scenarios including vocal animals. By using commercial microphones it is possible to detect the behaviour of a group of animals, as it is possible to record their intra-species communication and classify their behaviour based on the link between a certain behaviour and vocalisation. Another use, also regarding birds, could be recognition of seagul activity/harassment in cities or airports near the sea.

A complete system, capable of incorporating the automatic recognition of behaviour, is a part of ongoing research.

## 5. Conclusion

It is possible to distinguish between landing, foraging and flushing behaviour based on acoustic information. Landing and flushing behaviours have similarities in their vocalisations, however the accuracy for classification was over 90% for all behaviours.

The SVM modeling has proven robust, with generalisation capabilities, as results from the two test strategies are comparable. The use of GFCC as features shows promising results, however another choice of constants might prove more useful for this specific classification task.

Automatic behaviour recognition could improve automatic scaring devices, as it makes it possible to evaluate performance and alter strategies. In this paper it is shown that acoustic information can be used in the task of automatic recognition of landing, foraging and flushing behaviour.

## Figures and Tables

**Table 1.** Description and duration of the recorded behaviours.

**Behaviour**	**Definition**	**Number of events**	**Duration**		
			**Day 1**	**Day 2**	**Total**
Landing	Multiple geese approach the field and land on the ground	Two single events	48 s	30 s	78 s
Foraging	Multiple geese stay on the ground and pick food from the field	Multiple events ^a^	90 s	60 s	150 s
Flushing	Some geese take off, and the rest of the foraging flock follow, leaving the field empty of geese	Two single events	12 s	15 s	27 s

a Two events with high audio fidelity was selected, as the duration of foraging data should not be large compared to the other behaviours.

**Table 2.** Confusion matrix obtained from the classification of the three behaviours, using SVM with a six dimensional feature vector and RBF kernel function. The bold numbers indicate correct classification. The samples are 100 ms audio sequences. The notation A/B refers to the notation Test A and Test B, described in the Section 2.7. A: Classification where data has been divided based on date; B: Classification where data has been mixed.

**Observed behaviour**	**Predicted behaviour**			**Total**
	**Flushing**	**Landing**	**Foraging**	
Flushing	**129/44**	5/10	16/2	150/56
Landing	28/10	**219/144**	53/4	300/158
Foraging	5/13	14/28	**581/261**	600/302

**Estimate**	162/67	238/182	650/267	

**Table 3.** Model performance for each behaviour classification. The same notation A/B as in Table 2 is used in this table.

**Behavior**	**Performance**		
	**Accuracy ^a^**	**Precision ^b^**	**Sensitivity ^c^**
Flushing	0.95/0:93	0:80/0:66	0:86/0:79
Landing	0:91/0:90	0:92/0:79	0:73/0:91
Foraging	0:92/0:91	0:89/0:98	0:97/0:86

a Ratio of correct predictions (both positive and negative) that were correct;

b Ratio between correct postive and incorrect postive predictions;

c Ratio of correct classifications (ratio between the bold numbers and total samples).
